# Environmental contaminants and food safety

**DOI:** 10.1186/1751-0147-54-S1-S5

**Published:** 2012-02-24

**Authors:** Agneta Oskarsson

**Affiliations:** 1 Department of Biomedical Sciences and Veterinary Public Health, Swedish University of Agricultural Sciences, SE-750 07 Uppsala, Sweden

## 

Food safety is threatened by numerous contaminants, which can originate from environmental pollution, such as toxic metals and organic halogenated compounds; chemicals used in the production of food, such as pesticides and veterinary drugs; contaminants formed during food production and cooking; contaminants arising from food packaging, or natural toxins in food. Consumers’ perceptions of food-related risks have recently been investigated in the 2010 Eurobarometer. The highest concern was reported for pesticides in fruit, vegetables and cereals, with 72% of the respondents being very or fairly worried. Somewhat fewer people were worried about residues like antibiotics and hormones in meat (70%), pollutants like mercury and dioxins (69%), food poisoning from bacteria (62%) or putting on weight (47%).

Adverse effects of environmental contaminants may be displayed as developmental toxicity and endocrine disruption, with fetuses and children being vulnerable target groups. One contaminant, which has attracted much attention, is bisphenol A (BPA) and there is a scientific controversy about the low-dose health risks of BPA. BPA is used in the production of polycarbonate plastics and epoxy resins. Human exposure is mainly from packaged food and beverages. BPA binds to estrogen receptors and also acts through other mechanisms on endocrine function. We have investigated effects of BPA on steroidogenic pathways in the human adrenocortical cell line H295R. Secretion of steroidogenic hormones and intermediates were affected at non-toxic levels of BPA. The effects were mediated by inhibition of CYP17 and CYP21 and expressions of steroidogenic genes were downregulated. This may be an additional mechanism behind the endocrine disruptive effects of BPA.

One of the greatest challenges in toxicology today is in predicting the risks associated with chemical mixtures. The exposure to contaminants via the diet occurs as a mixture rather than as individual compounds. Thus, food safety is to a high extent dependant on possibilities to predict risks from mixtures. The two models most frequently used are concentration addition and independent action. The main difference between the two models is the assumption of mode of action of the chemicals in the mixture. In concentration addition it is assumed that the chemicals work through a common mode of action and can be regarded as dilutions of each other. In independent action it is assumed that the chemicals act independently via different mode of actions and the mixture effect is predicted by the probabilities of response of the individual chemicals. We have investigated mixture effects of food-related chemicals on secretion of steroids in the human adrenocortical cell line. The results have been compared to the predicted effects from the two prediction models. In general the chemicals acted in an additive manner on secretion of hormones, which could be predicted by both models.

